# Pseudocysts of the jaw: a retrospective study of 41 cases from a single institution

**DOI:** 10.1186/s12903-023-02741-5

**Published:** 2023-02-11

**Authors:** Yahui Wang, Fan Tang, Zhiyong Li, Qianming Chen

**Affiliations:** Stomatology Hospital, School of Stomatology, Zhejiang University School of Medicine, Clinical Research Center for Oral Diseases of Zhejiang Province, Key Laboratory of Oral Biomedical Research of Zhejiang Province, Cancer Center of Zhejiang University, Hangzhou, 310006 China

**Keywords:** Jaw cysts, Bone cysts, Aneurysmal

## Abstract

**Objective:**

The purpose of this retrospective study was to investigate and compare the clinical, radiographic, pathological, pathogenesis, and therapeutic features of simple bone cysts (SBCs) and aneurysmal bone cysts (ABCs) of the jaw.

**Methods:**

35 patients with SBCs and 6 patients with ABCs who received treatment at the Department of Oral and Maxillofacial Surgery, Zhejiang University School of Medicine from 2017 to 2022 were followed up and reviewed retrospectively.

**Results:**

The study included 41 patients, accounting for 2.14% of all jaw pathologies, with 35 patients having SBCs and 6 patients having ABCs; their average ages were 26.63 ± 13.62 years and 17.83 ± 7.88 years, respectively. The prevalence of SBC and ABC did not differ significantly by sex. The mandible was the most vulnerable area to be involved. Only 5.71% (2/35) of patients with SBCs and 16.7% (1/6) of patients with ABCs reported histories of previous trauma in the same region of the pseudocysts. A total of 42.86% (15/35) of SBC cases and 66.67% (4/6) of ABC cases had malocclusions. The radiographic features of pseudocysts varied in shape, were associated with the root, and unilocular or multilocular. All patients had curettage with or without bone graft or substitute implantation, and recurrences did not occur in 94.29% (33/35) of SBC patients and 100% (6/6) of ABC patients after a mean follow-up time of 26.23 ± 15.47 months and 21.67 ± 19.75 months, respectively.

**Conclusions:**

Pseudocysts, including SBCs and ABCs, are benign osteolytic lesions without an epithelial lining that occur occasionally in the jaw, mostly in adolescents and young adults, and their incidence did not significantly differ by sex. The most vulnerable site of involvement is the mandible, and they are generally not overtly aggressive. Trauma has a less significant role in pseudocysts, but minor trauma, such as malocclusion, has the potential to influence pseudocyst development. The clinical presentation of pseudocysts lacks specificity, and most patients are asymptomatic and found incidentally during radiographs. Dental panoramic radiographs and CBCT cannot accurately distinguish between SBC and ABC, and the final diagnosis depends on pathological diagnosis. Curettage combined with bone grafting is currently the best treatment for both, with a 5.71% (2/35) recurrence rate for SBC and no recurrence found for ABC.

## Introduction

Pseudocysts are defined as pathological cavities without an epithelial lining and with clinical and radiological findings similar to those of true cysts, including simple bone cysts (SBCs), aneurysmal bone cysts (ABCs), and Stafne bone cavities. SBCs and ABCs were included in the section 'Neoplasms and other bone lesions' in the 1992 World Health Organization (WHO) classification of odontogenic and nonodontogenic cysts, but as their clinicopathology became better understood, these entities were found not to be true cysts or neoplasms, and they are now classified under the section 'Giant cell lesions and bone cysts' in the 2017 WHO classification. Stafne bone cavities have not been classified by the WHO [1].

SBC, also known as traumatic bone cyst, solitary bone cyst, haemorrhagic bone cyst, and idiopathic bone cavity, was initially described by Lucas in 1929 [2]. Although it is common in the long bones, humerus, and femur, it only accounts for 1–2% of all cysts in the maxillofacial region. The aetiology and pathogenesis of SBC are unknown, but they may be related to trauma, venous obstruction, dysplasia, and other factors [3–5]. It is frequently asymptomatic and identified by radiography by chance.

ABC is a relatively rare benign osteolytic lesion that was first described by Jaffe and Lichtenstein in 1942 [6]. It mostly affects the spine and metaphysis of long bones, especially the tibia and femur, with only 2% of cases affecting the jaw. 70% of ABCs are primary, resulting from the rearrangement of the USP6 oncogene on chromosome 17, and 30% of ABCs are secondary, resulting from intraosseous or subperiosteal haemorrhage induced by aberrant venous circulation, which stimulates osteoclasts and causes bone resorption and local remodelling [7]. ABC has a similar clinical presentation and imaging to SBC, but it has a more aggressive clinical behaviour than SBC [8].

Pseudocysts of the maxillofacial region are difficult to identify because of their insidious symptoms. Although they are not true cysts or tumours, they have the potential to destroy the jaw and cause severe damage. Therefore, we reviewed the clinical, radiological, histological, and surgical data of patients with jaw pseudocysts admitted to the Department of Oral and Maxillofacial Surgery, Zhejiang University School of Medicine, from March 2017 to March 2022. We discussed the pathogenesis, diagnosis, and updated management in the literature to provide clinicians with a sufficient understanding of pseudocysts, allow them to distinguish pseudocysts from other jaw lesions, and avoid any misdiagnosis and inappropriate treatment.

## Material and methods

The data of SBC and ABC in jaws admitted to the Department of Oral and Maxillofacial Surgery of the Affiliated Stomatological Hospital of Zhejiang University School of Medicine between March 2017 and March 2022 were collected. 1) Clinical data: sex, age, location, history of trauma, malocclusion, vitality of the tooth pulp involved in the lesion, and reason for consultation. 2) Radiographic data: shape, presence of bone-white lines, unilocular or multilocular, the association of tooth roots with lesions, relationship and location of the cyst to the inferior alveolar canal. 3) Surgical data: treatment approach, contents of the bone cavity and postoperative neurological symptoms. 4) Follow-up data: follow-up time and results. The study was approved by the Ethics Committee of the Affiliated Stomatological Hospital of Zhejiang University School of Medicine, and informed consent was obtained from both the adult participants and the parent(s)/guardian(s) of all participants under 16 years old.

## Results

### Epidemiology

Of 1919 records, this retrospective survey reported 41 cases of pseudocysts of the jaws, which accounted for 2.14% of all jaw pathologies. Of these cases, 35 (1.82%) were SBCs, and 6 (0.31%) were ABCs.

### Clinical and radiographic features

#### SBC

The clinical and radiographic features of SBC are shown in Table 1. Of the 35 patients with SBC, 15 were male and 20 were female, ranging in age from 10 to 55 years, with a mean age of 26.63 ± 13.62 years old, and 42.86% (15/35) of patients were younger than 18 years. A total of 88.57% (31/35) of cases occurred in the mandible, and 11.43% (4/35) occurred in the maxilla. A history of previous maxillofacial trauma in the same area of the injury was reported in 5.71% (2/35) of cases. Clinical examination revealed that 42.86% (15/35) of patients had malocclusions of varying degrees. A total of 31.43% (11/35) of cases were seen for jaw bulge with pressure pain in the area of the lesion, 20.00% (7/35) were seen for orthodontic requirements, and 48.57% (17/35) were for dental disorders in nonlesioned areas. 35 cases of SBCs affected 74 teeth, 17.57% (13/35) of which were dead and had undergone root canal treatment preoperatively. Preoperative cone beam computed tomography (CBCT) revealed radiolucent images in the jaws with lesions of variable magnitude, shape, and borders in all patients. The lesions were spherical or round in shape in 74.29% (26/35) of the patients and irregular in shape in 25.71% (9/35) of the patients. A white line of bone resembling an apical cyst appeared in 20.00% (7/35) of the patients. A total of 91.43% (32/35) of cases were unilocular, and 8.57% (3/35) of cases had multilocular changes resembling ameloblastoma.

**Table 1:** Clinical and radiographic features of SBC

SBC	Category	Cases	Ratio
Gender	Male	15	42.86%
	Female	20	57.14%
Age	≤ 18	15	42.86%
	> 18	20	57.14%
	Maxilla		
Location	Anterior maxilla	2	5.71%
	Posterior maxilla	2	5.71%
	Mandible		
	Anterior mandible	7	20.00%
	Posterior mandible	24	68.57%
	Ramus of mandible	0	0
History of trauma	Yes	2	5.71%
	No	33	94.29%
Malocclusion	Yes	15	42.86%
	No	20	57.14%
The vitality of the tooth pulp involved in the lesion (74 teeth)	Normal	61	82.43%
	Abnormal	13	17.57%
Reason for consultation	Orthodontic requirements	7	20.00%
	Dental disorders in nonlesioned areas	17	48.57%
	Clinical symptoms	11	31.43%
	Bone white lines		
Radiographic features	Yes	7	20.00%
	No	28	80.00%
	Shape		
	Round	26	74.29%
	Irregular	9	25.71%
	Room(s)		
	Single	32	91.43%
	Multiple	3	8.57%
	Resorption/displacement of the roots		
	Yes	18	51.43%
	No	17	48.57%
	Inferior alveolar canal involved		
	Yes	22	62.86%
	No	13	37.14%
Bone cavity contents	Empty	20	57.14%
	Bloody liquid	8	22.86%
	Soft tissue	7	20.00%
Postoperative neurological symptoms	No symptoms	35	100.00%
	Neurological symptoms	0	0
Follow-up results	No recurrence	33	94.29%
	Recurrence	2	5.71%

**Fig. 1:**
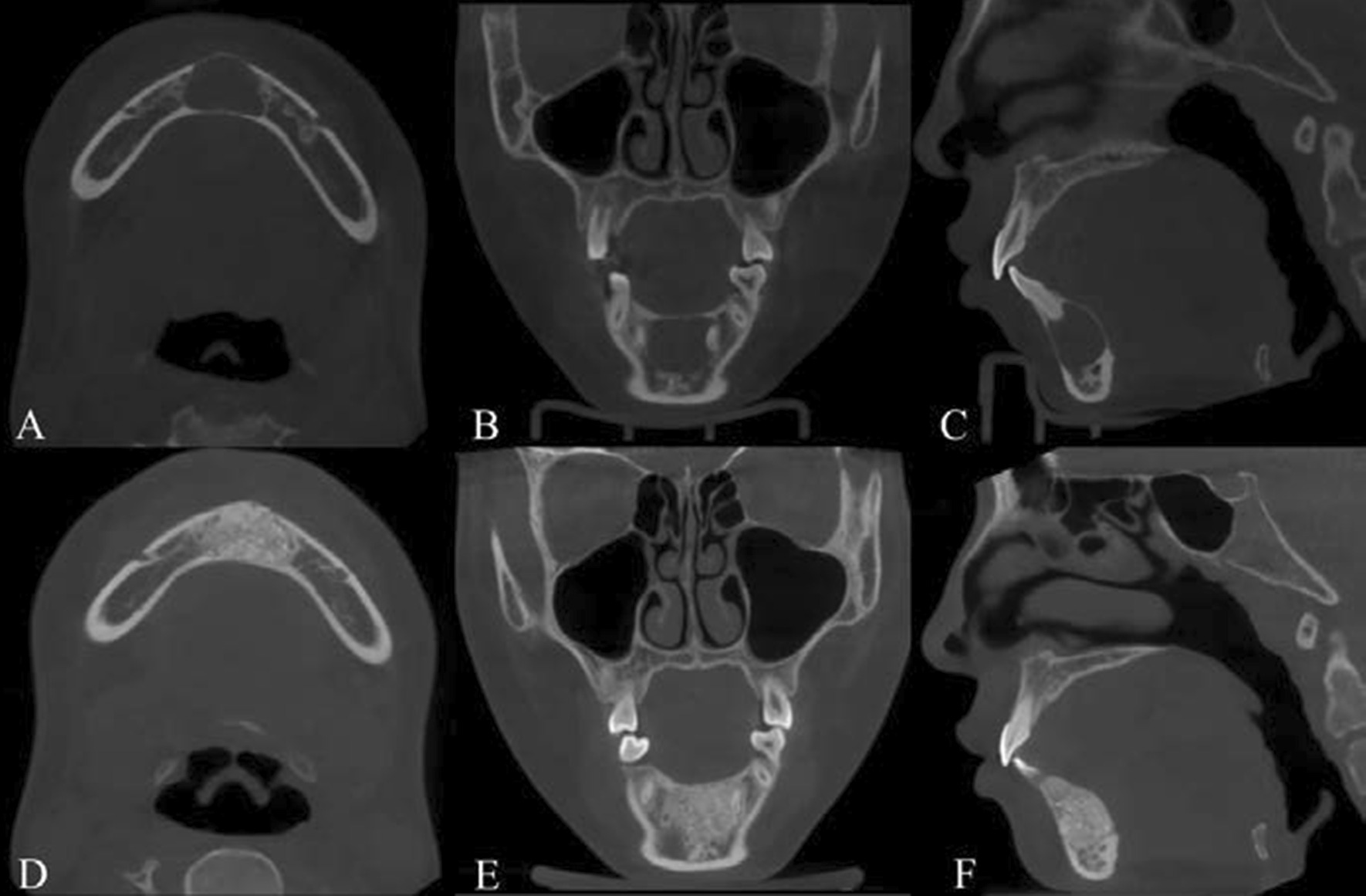
Radiographic features of SBC. **A**–**C** CBCT preoperatively for SBC. CBCT shows an isolated, round-like, unilocular radiolucent image of the lower anterior region with no bone-white line and no resorption and displacement of the adjacent root, mild expansion of the buccal cortex and thinning of the buccal and lingual cortex. **D**–**F** CBCT postoperation of SBC. CBCT shows dense bone filling within a proto-cyst in the lower anterior region

A total of 82.43% (61/74) of teeth were not affected by SBC, while 48.57% (17/74) of teeth were strongly associated with SBC and exhibited either roots in the lesion or roots that had resorbed and displaced. Cysts were involved the inferior alveolar canal in 62.86% (22/35) of patients. Furthermore, of the 88.57% (31/35) cases of SBC that occurred in the mandible, 70.97% (22/31) involved the inferior alveolar canal and showed a large range of radiolucent lesions with the inferior alveolar nerve located within the lesion, which was often found to be centrally located in the cavity during surgical exploration. A total of 29.03% (9/31) of SBC cases that did not involve the inferior alveolar canal showed a more limited range of radiolucent images close to the root of the tooth and located above the inferior alveolar canal. All patients underwent curettage combined with bone graft or substitute implantation. During surgical exploration, empty cavities were detected in 57.14% (20/35) of cases, a small amount of cystic tissue was detected in 20.00% (7/35) of cases, and bloody fluid was detected in 22.86% (8/35) of cases. We followed all patients for 7–56 months, with a mean follow-up of 26.23 ± 15.47 months. None of the patients reported any signs of inferior alveolar nerve damage. A total of 94.29% (33/35) of patients had no recurrence, and new bone production was detected by CBCT (Fig. 1); 5.71% (2/35) of patients had recurrence after 14 and 35 months, respectively.

#### ABC

**Fig. 2:**
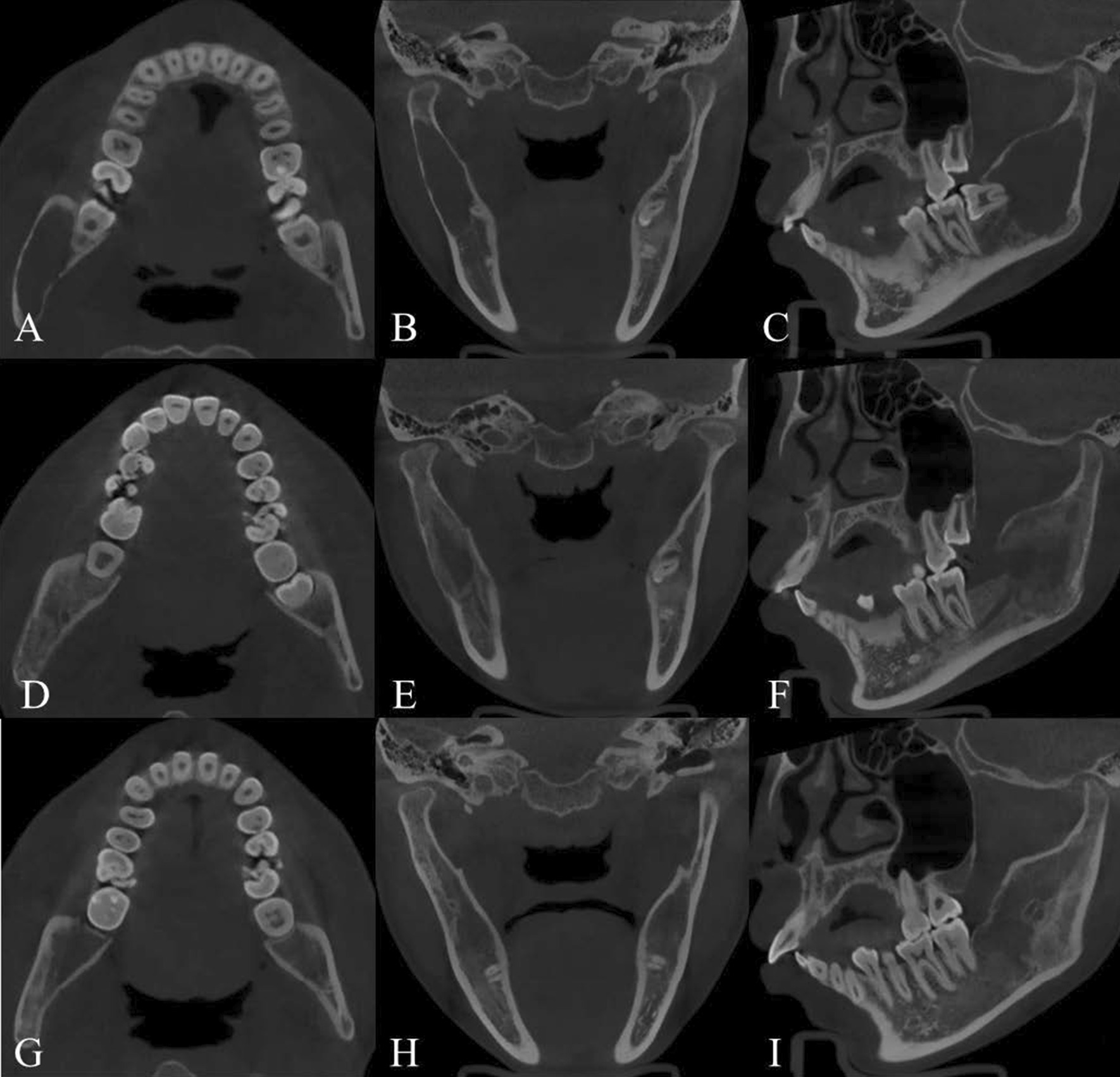
Radiographic features of ABC. **A**–**C** CBCT preoperatively for ABC. CBCT shows a large irregular, unilocular radiolucent image of the right mandibular body and ramus with thinning of the buccal and lingual cortex without bone-white lines and no significant resorption or displacement of the adjacent roots. **D**–**F** CBCT at 3 months postoperation of ABC. CBCT shows osteogenesis in the right mandibular body and ramus and thickening of the buccal and lingual cortex compared to the preoperative period. **G**–**I** CBCT 6 months postoperation of ABC. CBCT shows further osteogenesis in the right mandibular body and ramus with higher density than three months earlier and further thickening of the buccal and lingual cortex compared to the 3 months postoperation

The clinical and radiographic features of ABC are shown in Table 2. Of the 6 ABCs analysed, 50.00% (3/6) of patients were female, and 50.00% (3/6) were male. The age of patient ranged from 12–33 years old, with a mean age of 17.83 ± 7.88 years old, and 83.33% (5/6) of patients were younger than 18 years. All cases were in the mandible, with 66.67% (4/6) in the anterior region, 16.67% (1/6) in the posterior region and 16.67% (1/6) in the ramus of the region. A history of previous trauma in the same area of the injury was reported in only 16.67% (1/6) of cases. According to the clinical examination, 66.67% of patients (4/6) had malocclusions of varying degrees. Jaw bulges with mild pain were reported in only 16.67% (1/6) of cases, in 33.33% (2/6) of cases for orthodontic requirements, and in 50.00% (3/6) of cases for dental disorders in nonlesioned areas. Pulpal electrical vitality tests were normal in all 21 teeth involved in the ABC. Radiographic findings showed that all lesions were described as radiolucent lesions, with 66.67% (4/6) cases being irregular in shape, 50.00% (3/6) cases reported with white lines of bone, 83.33% (5/6) cases as unilocular lesions, and no root resorption or displacement in any of the 21 teeth involved in the ABC. Cysts were involved the inferior alveolar canal in 33.33% (2/6) of patients, and the ABCs that did not involve the inferior alveolar canal were located above the inferior alveolar canal in all 66.67% (4/6) cases. Similar to SBC, all patients underwent operations; empty cavities were detected in 33.33% (2/6) of cases, and bloody fluid was detected in 66.67% (4/6) of cases. All patients were free of recurrence, and new bone production was detected with a follow-up time of 8–56 months and a mean follow-up time of 21.67 ± 19.75 months. None of the patients reported any signs of inferior alveolar nerve damage (Fig. 2).

**Table 2:** Clinical and radiographic features of ABC

ABC	Category	Cases	Ratio
Gender	Male	3	50.00%
	Female	3	50.00%
Age	≤ 18	5	83.33%
	> 18	1	16.67%
Location	Maxilla	0	0
	Mandible		
	Anterior mandible	4	66.67%
	Posterior mandible	1	16.67%
	Ramus of mandible	1	16.67%
History of trauma	Yes	1	16.67%
	No	5	83.33%
Malocclusion	Yes	4	66.67%
	No	2	33.33%
The vitality of the tooth pulp involved in the lesion (21 teeth)	Normal	21	100%
	Abnormal	0	0
Reason for consultation	Orthodontic requirements	2	33.33%
	Dental disorders in nonlesioned areas	3	50.00%
	Clinical symptoms	1	16.67%
	Bone white lines		
Radiographic features	Yes	3	50.00%
	No	3	50.00%
	Shape		
	Round	2	33.33%
	Irregular	4	66.67%
	Room(s)		
	Single	5	83.33%
	Multiple	1	16.67%
	Resorption/displacement of the roots		
	Yes	0	0
	No	6	100%
	Inferior alveolar canal involved		
	Yes	2	33.33%
	No	4	66.67%
Bone cavity contents	Empty	2	33.33%
	Bloody liquid	4	66.67%
	Soft tissue	0	0
Postoperative neurological symptoms	No symptoms	6	100.00%
	Neurological symptoms	0	0
Follow-up results	No recurrence	6	100%
	Recurrence	0	0

### Histopathologic features

#### SBC

**Fig. 3:**
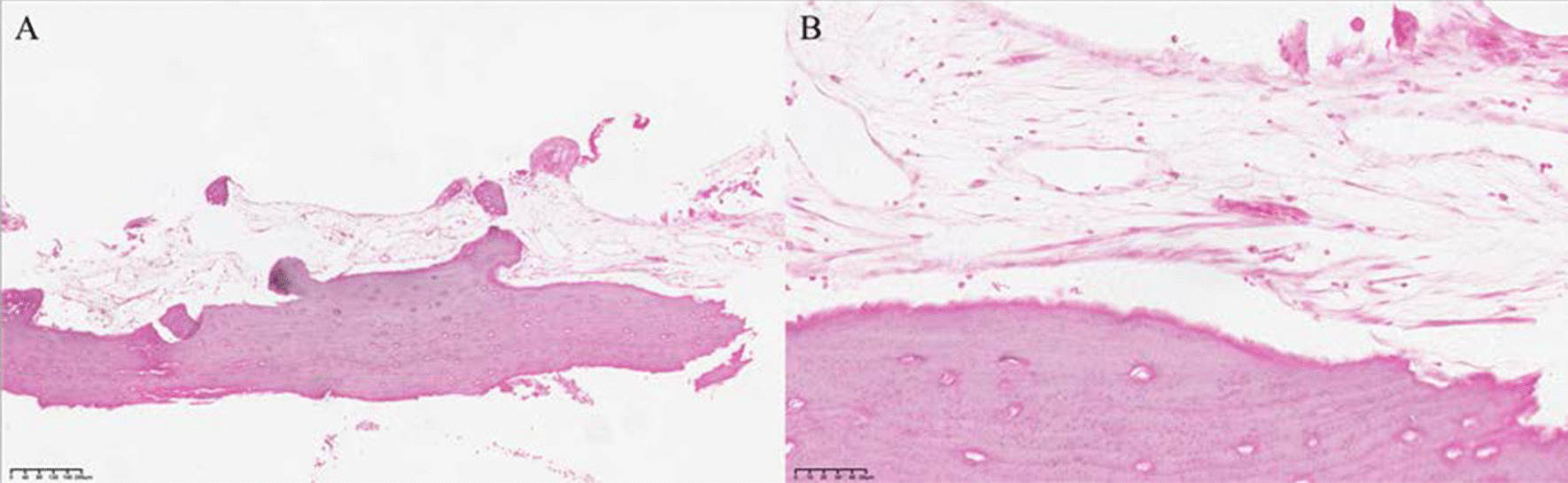
Histopathologic Features of SBC. **A** SBC H&E 10 × **B** SBC H&E 40 × Mature bone tissue is surrounded by a small amount of fibrous connective tissue, without lining epithelium, and ruptured blood vessels and a large number of red blood cells can be observed

Fibrous connective tissue proliferation without lining epithelium, massive vascularity around bone tissue, osteoblasts, bone-like matrix, and irregularly arranged bone trabeculae around new bone tissue can be observed in SBC lesions (Fig. 3).

### ABC

**Fig. 4:**
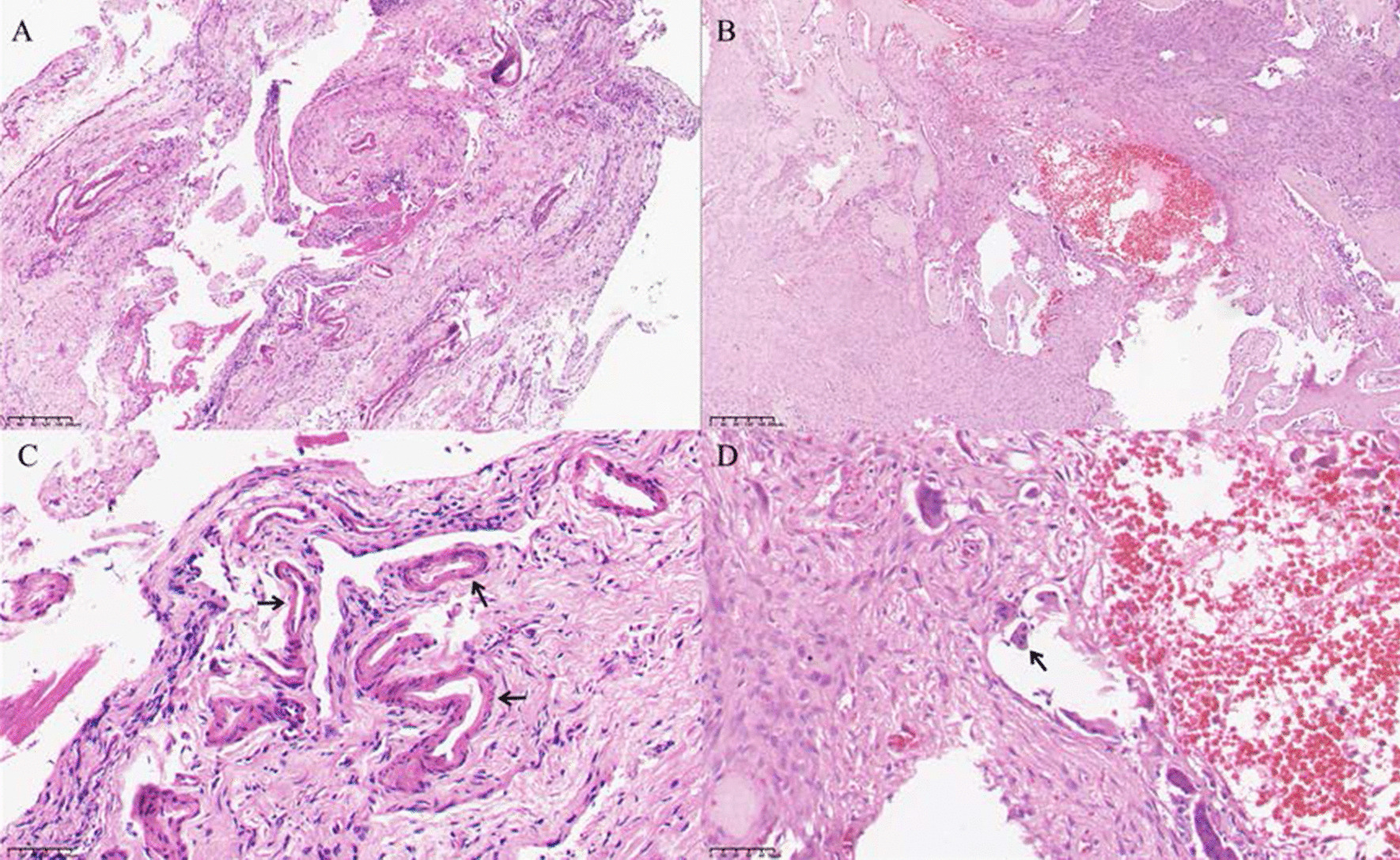
Histopathologic features of ABC. **A**, **B** H&E 10 × Fibrous connective tissue proliferation and a small amount of bone tissue without lining epithelium can be seen in ABC. **C**, **D** H&E 40 × Many hyperplastic, dilated, malformed small arteries and multinucleated giant cells and a large number of red blood cells can be observed in ABC. **C** Arrow: Malformed arteries. **D** Arrow: multinucleated giant cells

Many hyperplastic, dilated, malformed small arteries, along with multinucleated giant cells and a small amount of bone tissue, can be observed. Fibrous connective tissue proliferation without lining epithelium can also be seen in ABC lesions (Fig. 4).

## Discussion

### Epidemiology of pseudocysts

Our study found that SBC and ABC occurred more frequently in adolescents and young adults, with the mandible being the most involved region, which is consistent with the findings of many current studies [9–11]. SBC occurred more frequently in the posterior mandible, while ABC was more likely to involve the anterior mandible. However, in our retrospective study of a small sample of 41 cases, no significant difference in sex was detected.

### Etiopathogenesis of pseudocysts

The etiopathogenesis of SBC has not been extensively investigated thus far. Three theories were summarized by Harnet et al. [12]: 1) aberrant osseous growth, 2) the process of tumour degeneration, and 3) the trauma-haemorrhage theory. The most widely accepted theory is the last one, in which trauma leads to intramarrow haemorrhage and the subsequent formation of a haematoma, the pressure of which causes venous obstruction, producing intracapsular exudate. Furthermore, the enzymatic factors in the exudate promote bone resorption, leading to aseptic bone marrow necrosis. This mechanism also explains why SBC affects the mandible more than the maxilla. The bone cortex of the alveolar process of the maxilla is looser than that of the mandible because the blood supply of the maxilla is abundant and the bone cortex of the maxilla contains more foramina nutricium to facilitate the passage of microvessels. Compared to the maxilla, the mandible's bone cortex is thicker and denser, and its self-healing potential is weak [13]. Although the trauma-haemorrhage theory is now widely accepted, there is no proof that trauma is the aetiologic mechanism [14, 15]. Only 5.71% (2/35) of SBC patients in our study had reported histories of trauma, and all were maxillofacial impact injuries. As a result, we suspect that the effect of trauma on pseudocysts has been amplified. Nevertheless, minor oral and maxillofacial traumas that are rarely observed and remembered by patients, such as accidental biting on a hard item, might cause intramaxillary haemorrhage, resulting in venous blockage and SBC. Notably, 20.00% (7/35) of patients in our study were found to have SBC as a result of radiographs taken before orthodontic treatment, and all of them occurred in the mandibular anterior region. Furthermore, 42.86% (15/35) of SBC patients had malocclusion, which suggests that patients with malocclusion, especially those with chronic occlusal trauma such as overbite, mandibular crowding, heavy occlusion, occlusal interference, and premature contact, may also be at an increased risk of developing SBC. However, no studies have been performed to further investigate the relationship between SBC and malocclusion. More samples and collaboration with orthodontists will be needed to further explore whether the type of malocclusion is connected to the emergence of pseudocysts.

ABC can be classified as primary or secondary depending on the pathogenesis. Primary ABCs account for 70% of all ABCs. According to traditional theory, primary ABCs are thought to be produced by intraosseous or subperiosteal haemorrhage or other haemodynamic changes that cause blood vessels to dilate and rupture, thereby producing osteolysis. In the last decade, CDH11 and USP6 gene (TRE17) rearrangements have been observed in primary ABCs [16, 17]. The role of recurrent cytogenetic abnormalities in primary ABC is widely recognized. Secondary ABC is associated with a variety of lesions, including giant cell tumour of bone, chondroblastoma, osteoblastoma, mucinous fibroma of cartilage, osteosarcoma, osteochondrofibrodysplasia, and nonossifying fibroma, among other conditions [18]. For secondary ABCs in the jaw, the common accompanying lesions are ossifying fibroma and cemento-ossifying fibroma, which are rarely associated with malignant or metastatic bone lesions [13]. None of the ABCs were discovered to be related to other intramandibular diseases in our analysis of a limited sample of 6 individuals. Therefore, it has been suggested that primary ABC should be classified as a clonal neoplastic disorder rather than a pseudocyst [13]. However, due to the absence of an epithelial wall in either primary or secondary ABCs based on histopathological changes, the 2017 WHO classification still classifies ABCs as pseudocysts.

ABC is classified into 3 types based on histopathological features: vascular type, solid type, and mixed type. The vascular type is characterized by numerous engorged blood-filled sinusoids and loose fibrous connective tissue. A few blood arteries and strong fibrous connective tissue distinguish the solid type. The mixed type combines characteristics of the vascular and solid types. This presentation may be a transitory phase of the lesion [19]. The vascular variety accounts for 95% of all ABCs, and following surgery, severe haemorrhage and substantial bone destruction are common.

### Clinical manifestations of pseudocysts

According to our findings, 31.43% (11/35) of SBC patients were seen for clinical symptoms, such as pain and swelling, similar to a previous report [15]. However, only 16.67% (1/6) of ABC patients complained of swelling of the jaw. A systematic review of 44 cases of jaw ABC reported that most patients presented with rapidly progressive jaw swelling as a significant clinical feature [20]. Because of the nonspecific nature of their clinical symptoms, ABCs often need to be identified by imaging.

### Imaging manifestations of pseudocysts

Imaging examination is the most common method of examining intramaxillary lesions, and it can provide much information. Panoramic films can assess the morphology, density, and borders of the pseudocyst; CBCT can evaluate the bone in the lesion area, including the existence of cortical defects or bulges, the relationship with the root, and whether the cyst is unilocular or multilocular. On panoramic film or CBCT assessments of hard tissues, SBCs mostly appear as unilocular radiolucent images, which may have typical scalloping changes and the presence of osteosclerotic lines [21, 22].

ABCs mostly appear as a multilocular radiolucent or foamy images with osseous remodelling and cortical thinning [23]. Pseudocysts are easily misdiagnosed as other diseases due to the atypical features of their images. When they appear as unilocular radiolucent images, they need to be differentiated from central giant cell lesions, enamel cell fibromas, mural or unicystic ameloblastomas, and apical cysts or abscesses must be ruled out by clinical examination when the lesion is proximal to the root [24]. When multilocular changes are present, they need to be differentiated from ameloblastoma, keratotic cysts, and mucinous tumours [25–27]. Notably, preoperative imaging in 62.86% (22/35) of SBC cases and 33.33% (2/6) of ABC cases revealed cysts that were closely associated with the position of the inferior alveolar nerve canal, and the inferior alveolar canal appeared to be in the centre of the radiolucent lesions or near the cyst wall depending on the size of the cyst. SBCs and ABCs that did not involve the inferior alveolar canal were both located above the inferior alveolar canal and displayed a limited range of radiolucent close to the roots, primarily in the mandibular anterior and premolar regions. Extra care should be taken to preserve the inferior alveolar nerve during surgery on this portion of the cyst. When a true cyst or tumour involves the inferior alveolar nerve, the nerve may be stretched during intraoperative separation, resulting in nerve damage. Even when the cyst or tumour is difficult to separate from the inferior alveolar nerve, the nerve may be partially ligated as a last resort. In contrast, SBCs and ABCs are primarily cavities with a few soft tissues that resemble the cyst wall, and the surgical procedure is based on probing and scratching the surrounding bone wall, all of which preserve the inferior alveolar nerve. Therefore, the 41 patients in our study showed no signs of nerve injury during the postoperative follow-up.

On magnetic resonance imaging (MRI), SBCs are a centrally located, symmetrical, well-delineated unilocular or multilocular lytic lesions, and the contrast-enhanced T1WI of Gd-DTPA of SBC shows marked enhancement of the margin and slight enhancement of the inner part of the cyst cavity. This finding was not observed in true cysts with an epithelial lining [28, 29]. ABCs are expansile and destructive lesions, presenting as cystic lesions often with fluid‒fluid levels surrounded by a hypointense fibrous rim on all sequences [29, 30]. The appearance of fluid‒fluid levels is due to the stratification of the blood in the ABC, appearing as a strong signal in the upper layer, which is mainly plasma, and a low signal in the lower layer, which contains cellular debris [31]. Although panoramic radiographs and CBCT are sufficient for most intramaxillary cysts, MRI should be taken for patients with clinical suspicion of pseudocysts, and for highly vascularized lesions, such as ABCs and central haemangiomas. Failure to perform adequate preoperative assessment may lead to unexpected intraoperative crises. Some studies show ABC lesions are furnished by an external carotid artery or superficial temporal arteries; therefore, accurate assessment before the operation is essential to avoid uncontrollable bleeding [32, 33].

### Management of pseudocysts

Although cases of spontaneous regression of SBCs have been reported [34, 35], like most studies [15, 36], we advocate aggressive treatment for SBCs for the following reasons: 1. Aggressive treatment should be administered to individuals who exhibit clinical symptoms to relieve their pain after ruling out surgical contraindications. 2. Surgical treatment is advantageous to future treatment for people who need orthodontics. 3. For most patients who are asymptomatic but were diagnosed by chance on radiography, we attempted to prescribe conservative treatment by following them without treatment and performing routine imaging tests at 3, 6, 9, and 12 months. Although the patients did not complain of clinical symptoms, most of the cysts gradually increased in size, and few of them remained stationary or shrank on their own. Therefore, we usually recommend aggressive treatment in cases where cysts are found during the initial examination to prevent the enlargement of cysts, which can cause greater harm, such as the compression of the roots of the teeth, the compression of the nerve canals, or even pathological fractures. Currently, curettage combined with bone grafting or substitute implantation remains the mainstay of modern treatment for pseudocysts [37] and was the procedure applied in our study. Most patients received intraoperative bone grafting, but some of the patients who requested no bone grafting also showed bone regeneration on CBCT during the 3-, 6- and 9-month postoperative follow-up appointments. Today, several innovative bone replacement materials have flourished in the treatment of jaw defects due to the advancement of tissue engineering, including hydroxyapatite and β-tricalcium phosphate (β-TCP). β-TCP has also been successfully used in periodontal infrabony defect regeneration [38] due to its good biocompatibility and osteoconductivity [39]. Shuang Hu et al. [40] found that the application of β-TCP and biofilm exhibited good osteogenic effects to treat jaw cysts. As a result, the advancement of bone substitute material may hasten the healing of jaw defects while decreasing the risk of postoperative infection. Several studies have reported a high recurrence rate for curettage [9, 30]. The findings of our study revealed a recurrence rate of 5.71% (2/35) for SBCs and no cases of recurrence for ABCs, an outcome inconsistent with literature [41, 42]. This difference was most likely due to an insufficient sample size and short follow-up time. As a result, further research into predictors of pseudocyst prognosis should be carried out. With the increasing understanding of pseudocysts and the pursuit of minimally invasive surgical concepts, a variety of minimally invasive treatments for pseudocysts have arisen in recent years.

Hormone injections for SBCs have the advantages of being less invasive, easier to perform, and more convenient than surgery. In 1979, Scaglietti et al. [43] first used steroid hormones to treat SBC with positive results. The mechanism of this treatment may be the reduction of exudation and pressure within the cyst. Hashemi et al. [44] found that the efficacy of hormones to treat SBCs was inconclusive and that up to 12.5% of patients did not benefit from treatment, and 30% of patients experienced recurrence. Hormone injections are controversial in the treatment of SBC since they generally require multiple injections and can disturb the patient's hormone levels, especially in children who are still growing.

Since the healing of SBC is an osteogenic process, Lokiec et al. [45] first used the osteogenic properties of red bone marrow to treat SBCs in 1996 by injecting autologous red bone marrow extracted from the patient's iliac crest into the capsule cavity, with satisfactory results. Zamzam et al. [46] achieved over 80% efficiency in the treatment of SBC using autologous red bone marrow injection, and no significant complications were found. Therefore, autologous red bone marrow injection is considered a safe, effective, and low-risk treatment for SBCs. Wright et al. [47] compared the efficacy of autologous bone marrow transplantation and steroid injections for long bone SBC. At the end of the 2-year follow-up, the cure rate was higher in the hormone treatment group (42%) than in the bone marrow transplantation group (23%). However, conclusive findings on the efficacy of either of these methods for jaw SBCs are lacking, so future clinical trials are needed.

Decompression is widely recognized in the treatment of large cystic lesions of the jaw because of its minimally invasive nature and desired effect [48]. However, decompression is a long-term technique with a high failure rate that may necessitate second-stage surgery. ABCs have aberrant arterial and venous flow, and the cavity is filled with blood, which can lead to bleeding following decompression. Therefore, decompression is rarely used in the treatment of pseudocysts, especially for ABCs.

In recent years, radiation has been shown to be more effective in the treatment of ABCs, particularly in the treatment of recurring or large ABCs [49]. However, radiation therapy is not commonly employed as a treatment for ABCs due to the possible side effects of secondary malignancy, jaw necrosis, and radioactive stomatitis of the oral mucosa. The treatment of inoperable or recurrent ABC lesions of the vertebrae with radiation therapy doses of 26–30 Gy and procedures that minimize scatter has been shown to be effective and minimally toxicity [50], but doses or ranges for radiation therapy of jaw ABCs have not been established. In general, the role of radiotherapy in ABC treatment is questionable because most patients are young, even at the stage of skeletal growth. Therefore, the risk of serious adverse effects is considerable. However, local radionuclide therapy with phosphorus-32 chromic phosphate colloid under CT guidance has proven to be a safe and successful treatment option, and in one study, all lesions were cured without any complications [51].

Selective arterial embolization can be employed as a treatment for ABCs with a considerable risk of haemorrhage [52]. However, the use of selective arterial embolization may have unpredictable consequences due to the lack of identifiable blood supply vessels for the ABC on preoperative examination, and the vessels supplying the ABC also supply nearby vital tissues and organs. Therefore, it is rarely used to treat jaw ABCs.

Sclerotherapy works by disrupting the vascular endothelium, triggering the coagulation cascade and leading to thrombosis. Polidocanol is the sclerosing agent used for ABCs, and it is a relatively safe treatment with few major adverse effects. Rastogi et al. [53] reported a 97% cure rate for ABCs with the application of polidocanol. In a prospective study by Varshney et al. [54], sclerotherapy was shown to be as effective as intrafocal resection and had a lower recurrence rate. Polidocanol injection is a safe and simple procedure with a high success rate. Current evidence supports sclerotherapy as an initial treatment option for ABCs, but multiple injections are usually needed.

We summarize the clinical, radiographic, pathological, pathogenesis, and therapeutic features of two pseudocysts that occur in the jaws, as shown in Table 3.

**Table 3:** Comparison of SBC and ABC

	SBC	ABC
Gender	Male preferred	Female preferred
Age	< 20 years old	< 30 years old
Location	Most in long bones, rare in jaws	
Clinical Symptoms	Mostly asymptomatic	Swelling and pain
Radiographic features	X-rays and CBCT: unilocular preferred MRI: fluid‒fluid level is rare	X-rays and CBCT: multilocular preferred MRI: fluid‒fluid level is common
Histopathologic Features	Fibrous connective tissue proliferation without lining epithelium, massive vascularity, osteoblasts, bone-like matrix, and bone trabeculae can be observed	Fibrous connective tissue proliferation without lining epithelium, many hyperplastic, dilated, malformed small arteries, multinucleated giant cells, and bone tissue can be observed
aetiology and Pathogenesis	1) Abnormality of osseous growth 2) Tumour degeneration 3) Trauma-haemorrhage theory	1) Primary ABC: DH11 and USP6 gene (TRE17) rearrangements 2) Secondary ABC: haemorrhage and/or a reactive process
Therapeutic method	1) Surgical excision 2) Autologous red bone marrow injection therapy 3) Hormone injection therapy	1) Surgical excision 2) Polydocanol sclerotherapy 3) Selective arterial embolization 4) Radionuclide ablation

## Conclusion

SBCs and ABCs are pseudocysts because they lack a lining epithelium, and they are uncommon in the jaw. The clinical and imaging presentation of pseudocysts often lacks specificity, and MRI may guide the diagnosis of ABCs. The aetiology and pathogenesis of pseudocysts remain unclear, particularly in those occurring in the jaw, and whether they are associated with malocclusion still needs to be studied in large samples. Although pseudocysts are less invasive, they may cause jaw defects with serious consequences if left untreated, and curettage combined with innovative bone graft materials, autologous red bone marrow and sclerotherapy injections may be promising treatment modalities for pseudocysts. As a result, a thorough understanding of the aetiology of pseudocysts, as well as quick and accurate diagnosis, is critical to increasing the cure rate of pseudocysts. In the future, multicentre, large-sample studies will be required to further investigate the outcomes of SBC and ABC treatment and actively seek prognostic predictors.

## Data Availability

The datasets used and/or analyzed during the current study are available from the corresponding author on reasonable request.

## Abbreviations

SBC: Simple bone cyst
ABC: Aneurysmal bone cyst
WHO: World Health Organization
CBCT: Cone beam computed tomography
MRI: Magnetic resonance imaging
β-TCP: β-Tricalcium phosphate
